# Thermal and Dielectric Properties of Wolfberries as Affected by Moisture Content and Temperature Associated with Radio Frequency and Microwave Dehydrations

**DOI:** 10.3390/foods11233796

**Published:** 2022-11-24

**Authors:** Shunqin Bai, Li Liu, Haibo Yu, Xiangyu Guan, Rui Li, Lixia Hou, Bo Ling, Shaojin Wang

**Affiliations:** 1 College of Mechanical and Electronic Engineering, Northwest A&F University, Yangling District, Xianyang 712100, China; 2 School of Mechanical Engineering, Qinghai University, Xining 810016, China; 3 Department of Biological Systems Engineering, Washington State University, Pullman, WA 99164-6120, USA

**Keywords:** thermal properties, dielectric properties, penetration depth, true density, wolfberries

## Abstract

Knowledge of the thermal and dielectric properties of wolfberries is essential for understanding the heat transfer and the interaction between the electromagnetic field (10–3000 MHz) and the sample during radio frequency (RF) and microwave (MW) drying. The thermal and dielectric properties of wolfberries were determined as influenced by moisture content from 15.1% to 75.2%, w.b.) and temperature from 25 to 85 °C. The results showed that as the moisture content increased from 15.1% to 75.2% (w.b.), the true density of wolfberries decreased, but the specific heat capacity and thermal conductivity increased with increasing temperature and moisture content. The dielectric properties (DPs) of wolfberries decreased with increasing frequency from 10 to 3000 MHz. The dielectric constant increased with increasing temperature at lower a moisture content (below 45% w.b.) but decreased with increasing temperature at a high moisture content (above 60% w.b.). The cubic and quadratic polynomial models (*R*^2^ = 0.977 − 0.997) were best for fitting the dielectric constant and loss factor at four representative frequencies of 27, 40, 915, and 2450 MHz, respectively. The penetration depth increased with the decreased frequency, temperature, and moisture content, and was greater at RF frequencies than MW range, making the RF heating more effective for drying bulk wolfberries. These findings offered essential data before optimizing RF or MW dehydration protocols for wolfberries via computer simulation.

## 1. Introduction

The wolfberry (*Lycium barbarum* L.), also known as the goji berry, has been used in tonic food with traditional folk herbal medicine in China for more than 4000 years due to their abundant bioactive substances and medical functions [1]. Wolfberries have received increasing attention in recent years, and its utility values have gradually been discovered. A large number of studies have confirmed that the wolfberry is an effective supplement in the prevention of diseases, such as fatty liver, cancer, and cardiovascular diseases. In addition, the wolfberry has the effect of regulating immune system functions, blood lipids, and blood glucoses [2,3,4,5,6]. However, due to a high moisture content (MC), around 80% wet basis (w.b.), and tender tissue, fresh wolfberry is highly susceptible to softening rapidly and having a short shelf life. Thus, the wolfberry is usually consumed and preserved after drying.

As an important part of the processing of wolfberries, the drying process can reduce the water activity in wolfberries, effectively inhibiting active bacteria or enzymes, and improve the storage stability of wolfberries. Solar drying is the conventional dehydration technology for wolfberries. This method is highly affected by the uncertainty of the weather and is also easily contaminated with bacteria and dusts [7]. Hot air-drying transfers heat from the surface to the food center through thermal convection or conduction, resulting in extremely long drying time with lower heating efficiency because of the small thermal diffusivity of the products. The long exposure of fruits to high drying temperatures may cause a substantial degradation in product quality attributes [8]. Freeze-drying and vacuum-drying can better preserve nutrients and qualities by reducing the sublimation or boiling point. However, due to the requirement of low temperature or low pressure, the energy consumption and processing costs of freeze- or vacuum-drying are higher than those of other methods [9]. Recently, dielectric heating (DH) technologies, consisting of radio frequency (RF) and microwave (MW) treatments, have gained increasing interest in both industry and academia due to the unique rapid and volumetric heating. DH is generated within agricultural products by generated molecular friction due to dipole rotation and ionic movement [10,11]. As a result, RF and MW drying can shorten drying times, and improve the nutritional and sensory qualities of fruits compared to conventional methods. Therefore, DH has been investigated as a potentially safe and high efficiency drying method for many agricultural products, such as kiwifruits [12], carrots [13], mangoes [14], hazelnuts [15], mushrooms [16], apples [17], and walnuts [18].

However, heating non-uniformity remains a major issue in developing effective DH treatments for drying and other applications [19]. The performances of RF and MW drying are dependent on the thermal or dielectric characteristics of agricultural materials. A comprehensive understanding of the thermal and dielectric properties of wolfberries are necessary for improving DH performances and optimizing the drying protocols using computer simulation [20]. Thermal properties (TPs, containing true density, thermal conductivity, and specific heat capacity) could affect the absorption and conduction of thermal energy [21]. Thermal conductivity refers to rates of heat transfer within samples, while specific heat capacity determines the heat quantity that is gained or lost by a unit mass of materials to fulfil a unit variation of temperatures. During DH heating, the heating rate of samples increases with decreasing density and specific heat capacity when heat loss is ignored [21]. DPs (${\varepsilon =\varepsilon }^{\prime }-{j\times \varepsilon }^{\prime \prime }$) including the dielectric constant (ε′) and loss factor (ε″), describe the capability of samples for storing energy in electric fields and transforming electrical energy into thermal energy, respectively [22]. Meanwhile, *j* indicates the imaginary unit ($j=-1^{0.5}$). Since the MC, temperature, or frequency can affect the TPs and DPs of the samples [23,24,25,26], it is vital to systematically study the relevant properties of wolfberries so as to efficiently develop RF and MW drying methods.

The thermal and dielectric characteristics are found on numerous agricultural materials over various MCs, temperatures, or frequencies in drying [27,28,29], disinfesting [20], and pasteurization [30]. Mao et al. [25] indicated that the increased MC from 4.21% to 16.23% (w.b.) at temperatures from 20 to 80 °C resulted in the increased specific heat capacity and thermal conductivity of walnut kernels. Similar trends can be found in various fruits such as mango [31], jackfruit [32], and strawberry [33]. For DPs, Zhou et al. [28] determined the increased dielectric properties of kiwifruit that were caused by increasing the MC from 19.8% to 79.6% (w.b.) and decreasing the frequency from 3000 to 10 MHz. Their results also showed that increasing the sample temperature from 20 to 80 °C under high MCs slightly caused a decreased dielectric constant. Although there are various studies on the effects of MC and temperature on the DPs of fruits at RF or MW frequencies [28,34,35,36], to our knowledge, the data of the thermal or dielectric properties of wolfberries are still unavailable. Meanwhile, as a heat-sensitive fruit, the thermal properties for wolfberries are important to understand the amount of heat that is required in the drying process. Dielectric properties are also indispensable for the setting of drying parameters in the computer simulation and control of the drying process. Therefore, systematic research on thermal and dielectric properties of wolfberries is still needed to develop effective DH drying treatments.

The goals of this study were: (1) to detect TPs of wolfberries with five MCs (15%, 30%, 45%, 60%, and 75% w.b.) at four temperatures from 25 to 85 °C; (2) to determine DPs of wolfberries in the frequency range of 10–3000 MHz, five MC levels, and four temperatures (25, 45, 65, and 85 °C); (3) to establish regression equations fitted for the TPs and DPs of wolfberries at four representative frequencies (27, 40, 915, and 2450 MHz) as a function of temperature and MC; and (4) to estimate the penetration depth of dielectric power in wolfberries under different temperatures and MCs at these four specific frequencies that are widely used in DH implementations.

## 2. Materials and Methods

### 2.1. Sample Preparation

Wolfberry ‘Ningqi No.1′ was picked from a farm in Shizuishan, Ningxia, China. Fresh wolfberries were transported to the laboratory by cold-chain immediately after being harvested. To avoid possible infection by bacteria or fungi, the fresh samples were checked carefully to remove defective fruits. After that, the wolfberries without mechanical damage on the surface, complete, and uniform in size (long axis = 19.64 ± 2.21 mm, short axis = 9.64 ± 0.91 mm) with a weight of 1.26 ± 0.15 g were chosen and stored in a 4 °C refrigerator for subsequent experiments. Before the experiments, the samples were removed from the refrigerator and washed in running water to remove dust and impurities from the surface, then they were spread on filter paper to remove residual moisture. The initial MC of the samples with 78.86 ± 0.78% (w.b.) was determined by the AOAC Official Method 934.06 [37].

Since the desired final moisture content for wolfberry drying is 13% (w.b.), which is regarded as a safe standard for long-term preservation [38], five MC levels of 75%, 60%, 45%, 30%, and 15% (w.b.) were chosen. For preparing samples with 5 different MC levels, the washed wolfberries were divided into 5 groups. Each group of samples were uniformly distributed in one layer on PP trays (230 × 180 × 20 mm^3^) and placed on the metal tray of a heat pump dryer (WRH-100TB1, Guangdong Weierxin Industrial Co., LTD., Guangzhou, China). The temperature and relative humidity were 60 °C and 20%, respectively, in heat pump drying. The sample weight loss was determined every half hour by taking the tray out of the drying cavity and weighing it using an electronic balance (LT1002B, Changshu Tianliang Instrument co., LTD., Changshu, China) with an accuracy of 0.01 g. The sample MC was estimated according to the original mass, initial MC, and the mass after drying [39]. The drying procedure was finished when the required MC level of wolfberries s was achieved. The dried wolfberries were transferred from the drying chamber to the desiccator immediately for cooling, which can prevent the samples from absorbing moisture in the air. The final and actual MC levels of the samples were 75.1 ± 1.3%, 59.8 ± 1.8%, 45.3 ± 1.4%, 30.3 ± 1.1%, and 15.2 ± 1.2% (w.b.), which were determined according to the AOAC Official Method 934.06 [37].

### 2.2. Measurements of True Density

Before measuring the TPs and DPs of wolfberries, the true density of wolfberries at different MCs needs to be determined. To prevent displacement solution being absorbed by the samples, the liquid displacement method was used with toluene (C_7_H_8_) [25]. For each MC level, 3.325 ± 0.052 g wolfberries were randomly chosen and weighed (*w*, g) as an experimental group in triplicate. After that, the samples were placed into a Lee’s pycnometer with C_7_H_8_, and the volumes (*V*, cm^3^) that were occupied by the wolfberries were estimated. The equation to determine true density (*ρ*, g/cm^3^) can be expressed as:

(1) $$\rho =\frac{w}{V}$$

### 2.3. Measurement of Thermal Properties

A total of five groups of samples were pulped with a blender (DE-300g, Zhejiang Hongjingtian Co., Ltd., Jinhua, China) one by one after they fully cooled. Subsequently, each group was stored in airtight containers for TPs and DPs measurement separately. A thermal property analyzer (KD2 Pro, Decagon Devices, Inc., Pullman, WA, USA) with an SH-1 sensor was used to measure the thermal conductivity and specific heat capacity of wolfberries with five MC levels from 25 to 85 °C with the interval of 10 °C. The selection of this temperature range was based on the potential temperature for RF and MW drying. Based on the true density of wolfberries at each MC, wolfberries with a given weight were placed in a 10 mL beaker and immersed in a temperature-controlled water circulated bath (SC-15, Ningbo Xinzhi Biotechnology Co., LTD, Ningbo, China). The real sample temperature and TPs (thermal conductivity and specific heat capacity) were measured by a thermocouple (TMQSS-020-6, Omega Engineering, Inc., Norwalk, CT, USA) and the SH-1 sensor, respectively.

### 2.4. Measurement of Dielectric Properties

The measurement system consisted of an open-ended coaxial probe system (85070E-020), an impedance analyzer (E4991B-300), a temperature-controlled water circulated bath (SC-15, Ningbo Xinzhi Biotechnology Co., LTD, Ningbo, China), and a computer that was installed with dielectric software (85070E, Keysight Technologies Co., Ltd., Palo Alto, CA, USA), which was applied to evaluate DPs (dielectric constant and loss factor) of wolfberries with five MC levels. The details about the calibration and procedures of the DPs system could be found in Li et al. [26]. As the steps mentioned in Section 2.3, pulped samples with five MC levels were placed into the 25 mL beaker before measurement. The thermocouple with an accuracy of ± 0.5 °C was inserted into the sample center with depth of 20 mm. Measurements of the wolfberries’ DPs were performed at each given temperature. For each MC, the range of target temperatures was from 25 to 85 °C with an interval of 20 °C. The selected measurement range of frequencies was from 10 to 3000 MHz. When the wolfberries reached the target temperature, this coaxial probe was firmly affixed to the top surface of the wolfberries to detect DPs. The polynomial model for the regression analysis of DPs at 4 frequencies (27, 40, 912, and 2450 MHz) could be expressed as [40]:

(2) $$\varepsilon^{\prime }\mathrm{or}\;\varepsilon^{\prime \prime }=\alpha_{0}+\alpha_{1}T+\alpha_{2}M+\alpha_{3}TM+\alpha_{4}T^{2}+\alpha_{5}M^{2}+\alpha_{6}T^{2}M+\alpha_{7}TM^{2}+\alpha_{8}T^{3}+\alpha_{9}M^{3}$$

where ε’ and ε’’ are the dielectric constant and loss factor of wolfberries, respectively. α_0–9_ are regression coefficients. M and T represent MC and temperature, respectively. According to results of the analysis of variance (ANOVA), the term was removed when the corresponding *p* > 0.05. After ignoring the insignificant terms, the final regression equations were established again.

The penetration depth (*d_p_*, m) is defined as the distance where the power decreases to 1/e (e ≈ 2.718) of its amplitude entering the surface [27]. The equation to determine the penetration depth is described as:

(3) $$d_{p}=\frac{c}{2\pi f\sqrt{2\varepsilon \prime \left[\sqrt{1+{\left(\frac{\varepsilon \prime \prime }{\varepsilon \prime }\right)}^{2}}-1\right]}}$$

where *c* is the speed of light in free space (3 × 10^8^ m/s) and *f* is the frequency (MHz).

### 2.5. Statistical Analysis

The results were presented in averages ± standard deviations (SD) over three repetitions. The analysis of variance (ANOVA) and the least significant difference (*p* < 0.05) between different experimental values were performed using SPSS Statistics (21.0, SPSS Inc., Chicago, IL, USA). Origin (V2019b, Origin Lab Inc., Northampton, MA, USA) software was used to plot the graphs.

## 3. Results and Discussion

The data of the true densities for wolfberries with five MCs are listed in Table 1. The true density of wolfberries was reduced from 1.193 to 0.947 g/cm^3^ when the MC was raised from 15.2% to 75.1% (w.b.). This agreed with the trends that were reported by Zhu et al. [29], in which the true density of the chestnut kernel was reduced from 1430 to 1126 kg/m^3^ as the MC was raised from 10 to 60% (w.b.). The possible reason for this phenomenon could be that the increasing rate in wolfberries volume might be higher than that in weight due to the raised moisture [25]. Meanwhile, the true density (1.193 ± 0.019 g/cm^3^) of wolfberries with an MC of 15.2% was significantly greater than the value (0.947 ± 0.004 or 1.147 ± 0.012 g/cm^3^) of the sample with an MC of 75.1% or 45.3 ± 1.4%. Since significant differences (*p* < 0.05) were observed among wolfberries at various moisture levels, the true density of the samples should be maintained for the determination of TPs and DPs.

### 3.1. Thermal Properties of Wolfberries

**Figure 1:**
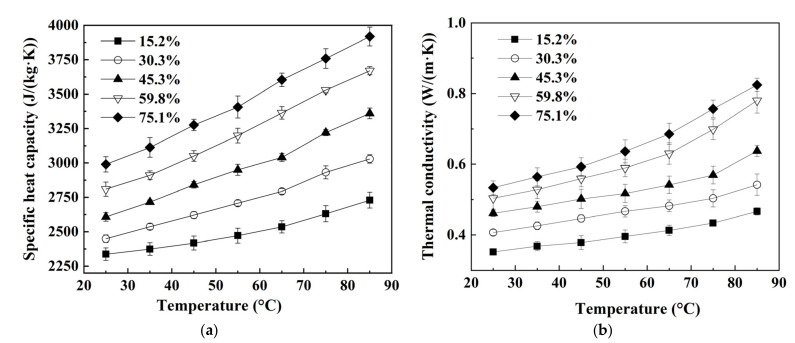
Moisture content and temperature-dependent specific heat capacity (**a**) and thermal conductivity (**b**) of wolfberries.

TPs at seven temperatures of wolfberries with five MCs are shown in Figure 1. The specific heat capacity and thermal conductivity of wolfberries both increased from 0.352 to 0.824 W/(m·K) and from 2337 to 3918 J/(kg·K) as the temperature increased from 25 to 85 °C at each MC, respectively. Similar results are also reported on watermelon seeds [39] and peanut kernels [41]. These two TP parameters increased with increasing MC of wolfberries. The results were consistent with those in the previous study that was reported by Mao et al. [25], who found that these two parameters of walnut shells also increased with increasing MC levels. On the one hand, when the MC of the sample was high, the specific heat capacity of the wolfberry was large due to the high specific heat capacity of water. On the other hand, because of the large volume of high-moisture materials with high mobility of water, the thermal conductivity of wolfberries was large [42]. Therefore, as the MC of the sample decreased during RF drying, the heating rate also decreased due to the reduced RF energy absorption.

### 3.2. Dielectric Properties of Wolfberries

#### 3.2.1. Frequency-Dependent DPs

**Figure 2:**
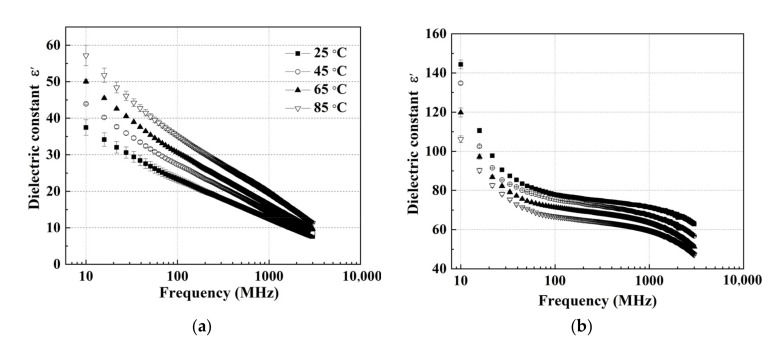
Frequency-dependent dielectric constant (*ε*′) of wolfberries with a moisture content of 15.2% (**a**) and 75.1% (w.b.) (**b**).

**Figure 3:**
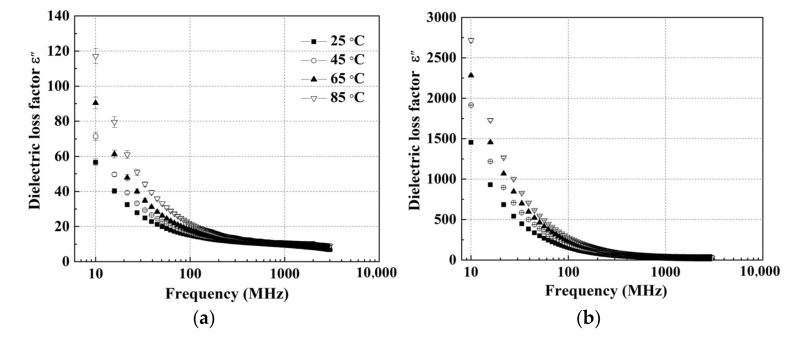
Frequency-dependent dielectric loss factor (*ε*″) of wolfberries with a moisture content of 15.2% (**a**) and 75.1% (w.b.) (**b**).

Figure 2 and Figure 3 show the DPs of wolfberries with an MC of 15.2% and 75.1% (w.b.) at the indicated temperatures (25 to 85 °C) over the frequency range from 10 to 3000 MHz. The DPs decreased with increasing frequency at any given temperature, which was consistent with the trends in the DPs of mango [31], jackfruit [32], and strawberry [33]. For the wolfberries in MC of 15.2% (w.b), the dielectric constant decreased sharply at each temperature over the frequency range from 10 to 3000 MHz (Figure 2a). However, for the sample with MC of 75.1%, the dielectric constant decreased with increasing frequency and the decrease was more obvious at lower frequencies under any temperature (Figure 2b). For example, when the frequency increased from 10 to 3000 MHz at 45 °C, the dielectric constant decreased rapidly from 134.75 to 76.77 at 10–80 MHz and decreased slowly from 76.77 to 56.70 at 80–3000 MHz. Meanwhile, the effect of frequency on the dielectric constant of wolfberries was more significant at high MCs. For instance, when the frequency increased from 10 to 300 MHz at 65 °C, the dielectric constant of the wolfberries decreased by 26.37 at an MC level of 15.2% w.b., while 51.69 at an MC level of 75.1% (w.b.). A possible reason for this discrepancy was the dispersion of water molecules in samples with a high MC [43]. Unlike the curve for the dielectric constant of walnut kernel [25], the value of wolfberries did not show a local maximum point. This was because the contribution of ionic conductivity in wolfberries could be greater than that of free water relaxation. The loss factor increased with increasing temperature at any frequency [25,44]. Similar trends for increased DPs with increasing temperature could also been found in kiwifruit [28], almond kernel [26], and peanut kernel [41]. In addition, as shown in Figure 3, the dielectric loss factor of samples with two MC levels dropped less at high frequencies than at low frequencies for any temperatures. Similar to the trend of the dielectric constant, the loss factor of wolfberries with high MC decreased more than that in low MC. The results were consistent with the DPs of kiwifruit [28], tuna [45], and chestnut flour [29]. At low frequencies (10–100 MHz), the ionic conduction in wolfberries contributed to the fact that DPs decreased rapidly with increasing frequency, while the increasing water relaxation changed the DPs slightly with increasing frequency (100–3000 MHz) [46].

#### 3.2.2. Moisture Content- and Temperature-Dependent DPs

**Figure 4:**
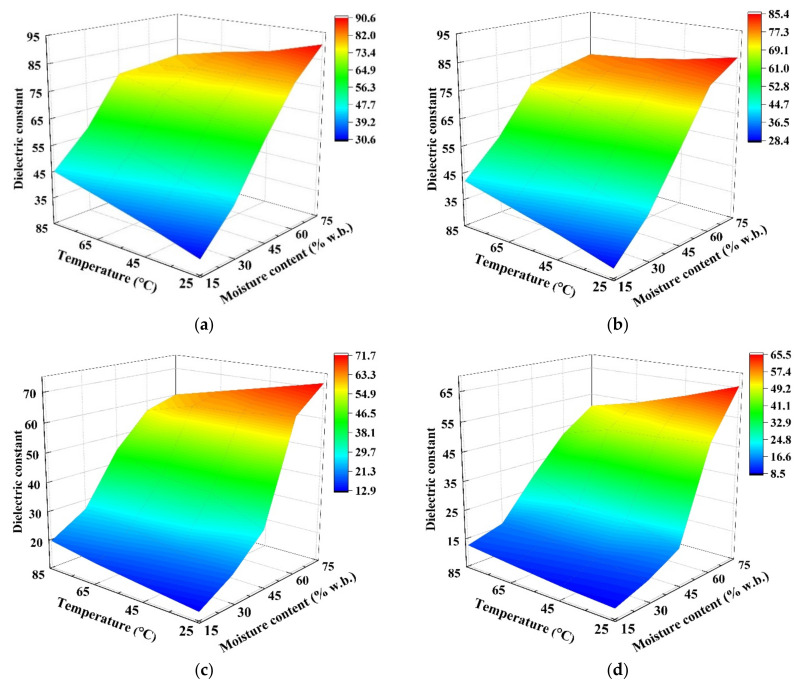
Moisture- and temperature-dependent dielectric constant (*ε*′) of wolfberries at 27 (**a**), 40 (**b**), 915 (**c**), and 2450 MHz (**d**).

**Figure 5:**
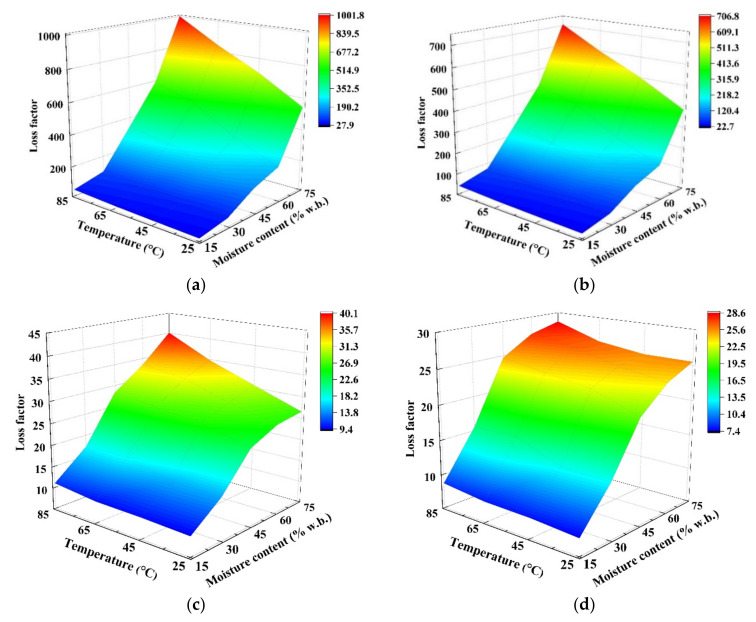
Moisture- and temperature-dependent dielectric loss factor (*ε*″) of wolfberries at 27 (**a**), 40 (**b**), 915 (**c**), and 2450 MHz (**d**).

The effects of MC level (15.2–75.1% w.b.) and temperature (25–85 °C) on the DPs of wolfberries at four frequencies of 27, 40, 915, and 2450 MHz are shown in Figure 4 and Figure 5. At the same temperature, the DPs decreased with decreasing MC levels in each frequency. On the one hand, since the water molecules in the wolfberries with high MC levels were arranged in a multilayer structure, both the ionic solubility and water dipole mobility were improved as compared with samples in low moisture levels [25]. On the other hand, Zhou et al. outlined that the mobility of water dipoles decreased, causing a reduction in the loss factor as drying progressed [28]. Similar results were observed in DPs of pineapple [47], potato [48], and kiwifruit [28].

As shown in Figure 5, the dielectric loss factor increased sharply with increasing temperature at high MCs (above 60% w.b.), especially at RF frequencies, while it increased slightly with increasing temperature at low MCs (below 45% w.b.). The possible reason for this phenomenon was that the ionic conductivity increased with decreasing biomaterials’ viscosity [49]. As the moisture content decreases, the free water is converted to bound water, the molecular viscosity increases, and the movement and diffusion of water molecules become difficult, so the loss factor decreases. Similar trends were also found in peanut kernels [27] and walnuts [25]. Also, as shown in Figure 4, the dielectric constant decreased slightly with increasing temperature at high MC levels. Due to this phenomenon, when the moisture content of the sample exceeded a threshold value, the RF heating rate decreased with the increase of the MC levels [11]. The trends of DPs that were observed in this study were similar to previous results that were observed in kiwifruit [28]. In addition, compared to samples with low MC, the parts in the wolfberry sample with higher MC content would absorb more thermal energy transformed from RF or MW heating. This phenomenon promotes rapid heating, thereby increasing the evaporation of water from the samples during dielectric drying, which is commonly named as the ‘moisture levelling effect’ reported by Mao et al. [25]. Based on the above results, the uniformity of temperature and moisture distributions in the whole sample would be improved during the RF or MW drying [50].

#### 3.2.3. Regression Models

The developed regression models for DPs of wolfberry as influenced by temperature and MC at 27, 40, 915, and 2450 MHz are listed in Table 2. Equations (4), (6), (8), or (10), and Equations (5), (7), (9), or (11) were the regression models for the dielectric constant and loss factor of wolfberries, respectively. The quadratic polynomial models were best for fitting the dielectric loss factor, while cubic polynomial models were best for fitting the dielectric constant at four frequencies of 27, 40, 915, and 2450 MHz. The fitting curve was a good fit with the experimental data as coefficients of determination (*R*^2^) approached 1. The *R*^2^ values of the fitted polynomial models of DPs for wolfberries were 0.977–0.997, indicating that these models could be capable of precisely predicting their DPs under given temperature and MC levels at four frequencies. Zhou et al. [28] also indicated that the *R*^2^ of quadratic and cubic polynomial models had competent ability to fit the data of DPs in kiwifruits with different MC levels and temperatures. To determine whether independent variables had a significant effect on DPs and models, the results of the ANOVA are listed in Table 3 and Table 4. Due to the significance level of 0.0001 (*p* < 0.0001), it also shown that the equations fitted the experimental data well. For all models of DPs for wolfberries, the linear term of M had very strong effect on these models (*p* < 0.0001). Thus, these models could be used to describe the moisture- and temperature-dependent DPs of wolfberries in future computer simulations at the four specific frequencies (27, 40, 915, and 2450 MHz).

### 3.3. Penetration Depth

The *d_p_* that was estimated from the measured DPs of wolfberries at five MC levels, four temperatures, and four frequencies are listed in Table 5. With decreasing MC, temperature, and frequency, the *d_p_* of electromagnetic waves into the sample increased. For instance, the *d_p_* decreased from 30.74 cm to 4.50 cm at 65 °C and 27 MHz when the moisture content of wolfberries increased from 15.1% to 75.1% w.b. Similar trends of *d_p_* as influenced by these parameters have been found in previous studies [28,51]. Meanwhile, when the frequency decreased from MW frequency (2450 MHz) to RF frequency (27 MHz), the *d_p_* of wolfberries with a moisture content of 30.3% (w.b.) increased from 0.61 cm to 22.71 cm at 25 °C. Li et al. indicated that the volume heating uniformity of the sample was better during RF heating since the penetration depth of RF waves in the sample was greater than that of MW [26]. Therefore, RF drying would be used to improve heating efficiency in the future for designing drying beds or protocols for wolfberries with a large volume and high throughput.

## 4. Conclusions

The true density of wolfberries increased with reducing moisture content. The TPs of wolfberries were influenced by the temperature and moisture content. The DPs of wolfberries were largely affected by frequency, moisture content, and temperature. When the frequency was fixed, DPs decreased with decreasing moisture content and temperature. The quadratic and cubic polynomial models (*R*^2^ = 0.977–0.997) were best for fitting DPs at four frequencies of 27, 40, 915, and 2450 MHz, respectively. The penetration depth increased with decreasing moisture content, temperature, and one at RF range was greater than that at MW frequencies, making the RF energy more effective for drying bulk wolfberries. Further studies would be focused on optimizing the efficacious dielectric drying protocols for wolfberries using an appropriate sample thickness.

## Figures and Tables

**Table 1:** True densities (mean ± SD over three replicates) of wolfberries at five moisture contents.

Moisture Content (w.b.)	True Density (g/cm^3^)
15.2 ± 1.2%	1.193 ± 0.019 a *
30.3 ± 1.1%	1.166 ± 0.004 ab
45.3 ± 1.4%	1.147 ± 0.012 b
59.8 ± 1.8%	1.126 ± 0.042 b
75.1 ± 1.3%	0.947 ± 0.004 c

* Different lower-case letters indicate that the means are significantly different at *p* < 0.05 among different moisture contents.

**Table 2:** Regression equations of the dielectric properties of wolfberries at four representative frequencies as a function of moisture content (*M*, % w.b.) and temperature (*T*, °C).

Frequency (MHz)	Dielectric Properties	Equations
27	ε′ = 20.64 + 0.16*T* − 19.81*M* + 0.82*TM* + 330.70*M*^2^ − 1.77*M*^2^*T* − 227.30*M*^3^	(4)
	ε″ = 212.80 − 2.44*T* − 1295.00*M* + 13.38*TM* + 1962.00*M*^2^	(5)
40	ε′ = 25.10 − 0.14*T* − 82.66*M* + 0.83*TM* + 495.85*M*^2^−1.77*M*^2^*T*−356.02*M*^3^	(6)
	ε″ = 151.70 − 1.71*T* − 901.23*M* + 9.39*TM* + 1369.00*M*^2^	(7)
915	ε′ = −3.72 + 72.69*M* + 0.93*TM*−1.57*M*^2^*T* + 66.77*M*^3^	(8)
	ε″ = 1.54 − 0.04*T* + 54.95*M* + 0.33*TM* − 36.79*M*^2^	(9)
2450	ε′ = 30.99−276.90*M* + 0.91*TM* + 770.70*M*^2^−1.67*M*^2^*T*−438.21*M*^3^	(10)
	ε″ = −5.99 + 0.05*T* + 78.81*M* − 52.21*M*^2^	(11)

**Table 3:** Analysis of variance of regressed models of Equations (4), (6), (8), and (10) for wolfberries at four frequencies relevant to dehydration.

Variance and *R*^2^	27 MHz (Equation (4))		40 MHz (Equation (6))		915 MHz (Equation (8))		2450 MHz (Equation (10))	
	F-Value	*p*-Value	F-Value	*p*-Value	F-Value	*p*-Value	F-Value	*p*-Value
*T*	7.246	0.0226	13.608	0.0042	0.995	0.3420	0.737	0.4109
*M*	215.363	<0.0001	581.503	<0.0001	169.904	<0.0001	119.065	<0.0001
*TM*	73.438	<0.0001	160.598	<0.0001	14.337	0.0036	5.007	0.0492
*T* ^2^	0.001	0.9799	0.157	0.7004	0.063	0.8069	0.019	0.8922
*M* ^2^	41.620	<0.0001	116.886	<0.0001	0.693	0.4247	25.166	0.0005
*T* ^2^ *M*	0.123	0.7335	0.0001	0.9905	0.041	0.8444	0.001	0.9765
*TM* ^2^	12.091	0.0059	27.172	0.0004	6.431	0.0296	6.215	0.0318
*T* ^3^	0.125	0.7307	0.183	0.6776	0.013	0.9122	0.020	0.8896
*M* ^3^	9.251	0.0124	51.168	<0.0001	25.777	0.0005	11.137	0.0075
Model	191.722	<0.0001	420.048	<0.0001	91.154	<0.0001	68.785	<0.0001
*R* ^2^	0.994		0.997		0.988		0.984	

**Table 4:** Analysis of variance of regressed models of Equations (5), (7), (9), and (11) for wolfberries at four frequencies relevant to dehydration.

Variance and *R*^2^	27 MHz (Equation (5))		40 MHz (Equation (7))		915 MHz (Equation (9))		2450 MHz (Equation (11))	
	F-Value	*p*-Value	F-Value	*p*-Value	F-Value	*p*-Value	F-Value	*p*-Value
*T*	55.004	<0.0001	117.475	<0.0001	59.110	<0.0001	17.160	0.0010
*M*	789.362	<0.0001	1199.657	<0.0001	751.532	<0.0001	532.508	<0.0001
*TM*	73.609	<0.0001	73.709	<0.0001	25.173	0.0002	0.277	0.6070
*T* ^2^	0.077	0.7860	0.071	0.7938	0.050	0.8266	0.005	0.9459
*M* ^2^	99.675	<0.0001	98.608	<0.0001	20.259	0.0005	45.142	<0.0001
Model	297.693	<0.0001	297.904	<0.0001	171.225	<0.0001	119.018	<0.0001
*R* ^2^	0.991		0.991		0.984		0.977	

**Table 5:** Penetration depth of electromagnetic waves into wolfberries at different frequencies, moisture contents, and temperatures (*T*, °C).

Moisture Content (% w.b.)	*T* (°C)	Penetration Depth (cm)			
		27 MHz	40 MHz	915 MHz	2450 MHz
15.1	25	38.84 ± 0.73	30.68 ± 0.59	2.16 ± 0.01	0.85 ± 0.00
	45	34.36 ± 0.75	27.64 ± 0.42	2.13 ± 0.01	0.81 ± 0.02
	65	30.74 ± 0.87	25.21 ± 0.71	2.06 ± 0.01	0.79 ± 0.02
	85	25.51 ± 0.75	20.92 ± 0.57	1.99 ± 0.01	0.75 ± 0.01
30.3	25	22.71 ± 1.38	18.55 ± 1.15	1.74 ± 0.12	0.61 ± 0.04
	45	20.44 ± 0.24	16.83 ± 0.11	1.70 ± 0.06	0.60 ± 0.02
	65	18.70 ± 0.31	15.49 ± 0.26	1.56 ± 0.01	0.53 ± 0.00
	85	16.43 ± 0.12	13.73 ± 0.10	1.51 ± 0.00	0.51 ± 0.00
45.3	25	11.75 ± 0.19	9.85 ± 0.16	1.33 ± 0.05	0.46 ± 0.02
	45	9.60 ± 0.06	8.03 ± 0.04	1.31 ± 0.03	0.43 ± 0.01
	65	8.26 ± 0.01	6.87 ± 0.03	1.28 ± 0.02	0.42 ± 0.01
	85	7.52 ± 0.09	6.25 ± 0.09	1.26 ± 0.01	0.40 ± 0.00
59.8	25	9.86 ± 0.02	8.37 ± 0.02	1.65 ± 0.02	0.59 ± 0.01
	45	7.57 ± 0.09	6.32 ± 0.08	1.48 ± 0.01	0.56 ± 0.01
	65	5.97 ± 0.07	4.94 ± 0.06	1.31 ± 0.01	0.53 ± 0.00
	85	5.36 ± 0.18	4.42 ± 0.15	1.20 ± 0.04	0.48 ± 0.01
75.1	25	5.80 ± 0.03	4.80 ± 0.02	1.69 ± 0.01	0.63 ± 0.00
	45	4.97 ± 0.01	4.10 ± 0.01	1.45 ± 0.01	0.61 ± 0.00
	65	4.50 ± 0.02	3.69 ± 0.01	1.24 ± 0.01	0.56 ± 0.01
	85	4.09 ± 0.03	3.34 ± 0.03	1.06 ± 0.03	0.50 ± 0.01

## Data Availability

The datasets that were generated for this study are available on request to the corresponding author.
